# Generation and characteristics of human Sertoli cell line immortalized by overexpression of human telomerase

**DOI:** 10.18632/oncotarget.14985

**Published:** 2017-01-27

**Authors:** Liping Wen, Qingqing Yuan, Min Sun, Minghui Niu, Hong Wang, Hongyong Fu, Fan Zhou, Chencheng Yao, Xiaobo Wang, Zheng Li, Zuping He

**Affiliations:** ^1^ State Key Laboratory of Oncogenes and Related Genes, Renji-Med X Clinical Stem Cell Research Center, Ren Ji Hospital, School of Medicine, Shanghai Jiao Tong University, Shanghai 200127, China; ^2^ Department of Andrology, Urologic Medical Center, Shanghai General Hospital, Shanghai Jiao Tong University, Shanghai 200080, China; ^3^ Shanghai Institute of Andrology, Ren Ji Hospital, School of Medicine, Shanghai Jiao Tong University, Shanghai 200001, China; ^4^ Shanghai Key Laboratory of Assisted Reproduction and Reproductive Genetics, Shanghai 200127, China; ^5^ Shanghai Key Laboratory of Reproductive Medicine, Shanghai 200025, China

**Keywords:** human, Sertoli cell line, human telomerase, phenotypic characteristics, proliferation

## Abstract

Sertoli cells are required for normal spermatogenesis and they can be reprogrammed to other types of functional cells. However, the number of primary Sertoli cells is rare and human Sertoli cell line is unavailable. In this study, we have for the first time reported a stable human Sertoli cell line, namely hS1 cells, by overexpression of human telomerase. The hS1 cells expressed a number of hallmarks for human Sertoli cells, including SOX9, WT1, GDNF, SCF, BMP4, BMP6, GATA4, and VIM, and they were negative for 3β-HSD, SMA, and VASA. Higher levels of AR and FSHR were observed in hS1 cells compared to primary human Sertoli cells. Microarray analysis showed that 70.4% of global gene profiles of hS1 cells were similar to primary human Sertoli cells. Proliferation assay demonstrated that hS1 cells proliferated rapidly and they could be passaged for more than 30 times in 6 months. Neither Y chromosome microdeletion nor tumorgenesis was detected in this cell line and 90% normal karyotypes existed in hS1 cells. Collectively, we have established the first human Sertoli cell line with phenotype of primary human Sertoli cells, an unlimited proliferation potential and high safety, which could offer sufficient human Sertoli cells for basic research as well as reproductive and regenerative medicine.

## INTRODUCTION

Sertoli cells are essential for normal spermatogenesis which comprises the mitosis of spermatogonial stem cells, the meiosis of spermatocytes, and the spermiogenesis of haploid spermatids. As the only type of somatic cells within the seminiferous tubule of the testis, Sertoli cells provide critical structural and nutritional supports for male germ cells, including spermatogonia, spermatocytes and spermatids [1]. The tight junction, constituted by Sertoli cells, is the key structure of the blood-testis barrier (BTB) to ensure the stabilization of the microenvironment or niche of the testis. Tight junction proteins, e.g., zona occludens 1 (ZO1), claudin 11 (CLDN11) [2], and occluding (OCLN), are produced by Sertoli cells, and they play vital roles in controlling the function of BTB [3]. In addition to structural support, Sertoli cells play significant roles in promoting the self-renewal, differentiation, and apoptosis of spermatogonial stem cells via secreting a number of growth factors, including bone morphogenetic protein 4 (BMP4), stem cell factor (SCF), leukemia inhibitory factor (LIF), glial cell derived neurotrophic factor (GDNF), fibroblast growth factor 2 (FGF2), and epidermal growth factor (EGF) [1]. Consequently, any abnormality in the number and/or function of Sertoli cells can lead to spermatogenic arrest and eventual spermatogenesis failure [4–6]. Therefore, Sertoli cells have significant applications in reproductive medicine.

A number of lines have implicated that Sertoli cells could have important applications in regenerative medicine because of their great plasticity. Firstly, Sertoli cells could secrete insulin persistently after being genetically transformed [7], which may provide new therapeutic approach for diabetes. Secondly, Sertoli cells could be reprogrammed to the pluripotent stem cells and to morphologic, phenotypic and functional neural stem cells by transferring certain genes or transcription factors [8–10]. These studies illustrate that Sertoli cells might be utilized for treating neural disorders and other human diseases. Finally, Sertoli cells could be converted into Leydig cells after deletion of gene *Wt*1 (Wilms tumor 1) [11], suggesting that Sertoli cells could be used in the therapy for testosterone- or other hormones-deficiency disorders.

On the other hand, the application of human Sertoli cells has been handicapped due to the following factors: i) it is hard to obtain human testicular tissues to separate human Sertoli cells; ii) the isolation and purification of human Sertoli cells from testis tissues seem to be tedious and time-consuming; iii) the number of primary human Sertoli cells is rare; and iv) primary human Sertoli cells proliferate limitedly *in vitro*. Therefore, it is necessary to establish a human Sertoli cell line to obtain adequate human Sertoli cells for basic research to uncover the mechanisms underlying human spermatogenesis as well as for their applications in both reproductive and regenerative medicine. Currently, several germ cell lines in human or rodents and Sertoli cell lines in rodents have been established with either simian virus large T antigen (SV40-LTAg) or telomerase (TERT) [12–15]. However, human Sertoli cell line has not yet been available. In this study, we have for the first time reported a stable human Sertoli cell line, namely hS1 cells, via the overexpression of human telomerase (hTERT). This cell line possessed the phenotypic characteristics of primary human Sertoli cells and it had an unlimited proliferation potential and high safety, which could offer a sufficient source of human Sertoli cells for their use from the bench to the bed side.

## RESULTS

### Immortalization of human Sertoli cells

**Figure 1:**
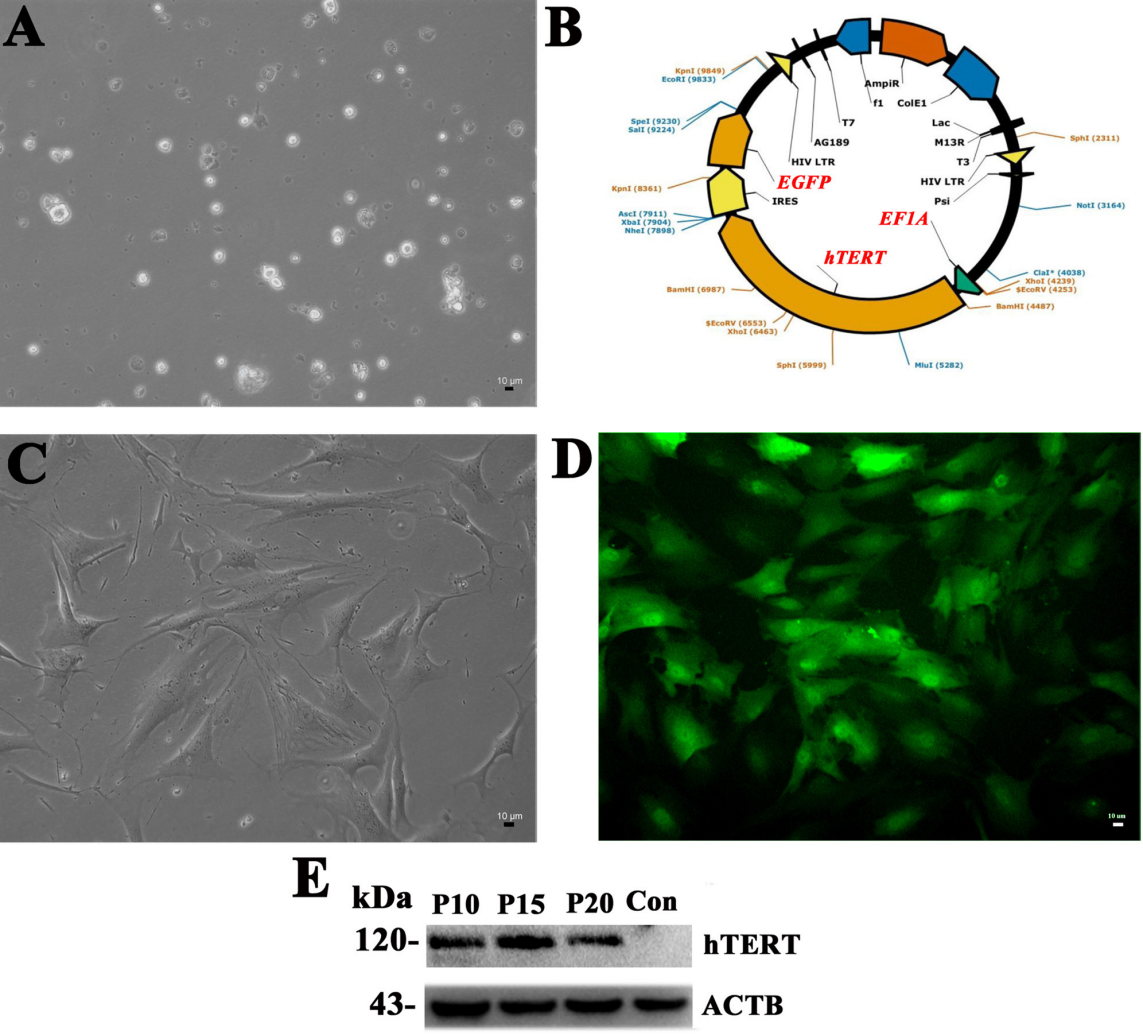
Immortalization of human Sertoli cells (**A**) Human Sertoli cells were freshly isolated from human testis tissues derived from OA patients by a two-step enzymatic digestion and differential plating. (**B**) The diagram showed the structure of lentivirus vector namely Lv-EF1A-hTERT-IRES-EGFP. (**C**) The isolated human Sertoli cells adhered to the culture dish. (**D**) EGFP-positive Sertoli cells infected with Lv-EF1A-hTERT-IRES-EGFP were sorted by FACS. Scale bars in A, C, D = 10 μm. (**E**) The expression of hTERT in the immortalized Sertoli cells at passage 10, 15, 20 and primary human Sertoli cells (the control). ACTB was used as a loading control of the proteins.

Human Sertoli cells were separated from testicular tissues of obstructive azoospermic (OA) patients with normal spermatogenesis using a two-step enzymatic digestion and followed by differential plating (Figure 1A, 1C), and they were infected with the lentivirus carrying *hTRET* by polybrene. The expression of hTRET was driven by the promoter of *EF1A* (Figure 1B), and *EGFP* was utilized as a reporter gene (Figure 1B). GFP-positive cells (the immortalized Sertoli cells), namely hS1 cells, were isolated and purified by FACS, and the EGFP was stably detected under a fluorescence microscope (Figure 1D). Western blots showed that the protein of hTERT was stably expressed in human Sertoli cell line at passage 10 (P10), P15, and P20 (Figure 1E). In morphology, the immortalized human Sertoli cells extended their cytoplasm with irregular nuclei under a phase-contrast microscopy.

### Phenotypic identification of human Sertoli cell line

**Figure 2:**
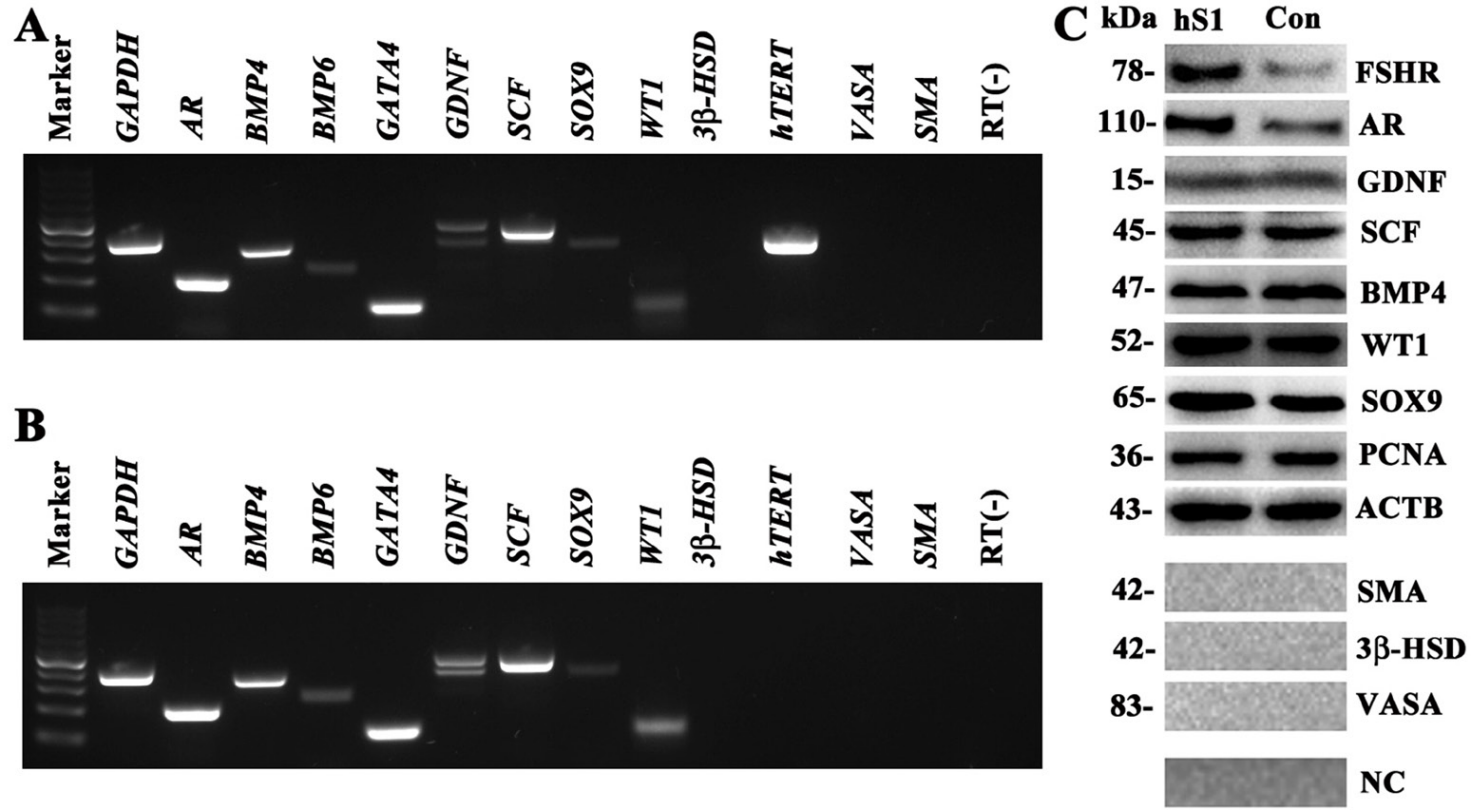
Phenotypic feature of the immortalized human Sertoli cells (**A–B**) RT-PCR showed the expression of *AR, BMP4, BMP6, GATA4, GDNF, SCF, SOX9*, *WT1, hTERT, 3β-HSD, SMA*, and *VASA* in the immortalized human Sertoli cells (A) and primary human Sertoli cell (B). *GAPDH* was used as a loading control of total RNA, and RNA sample without RT (RT-) but with PCR of *GAPDH* primers served as a negative control. (**C**) Western blot revealed the proteins of FSHR, AR, GDNF, SCF, BMP4, WT1, SOX9, PCNA, 3β-HSD, VASA, and SMA in the immortalized human Sertoli cells (hS1) and primary Sertoli cells (Con). ACTB was used as a loading control of proteins, while replacement of primary antibodies with PBS served as negative controls (NC).

To check whether the EGFP-positive cells were human Sertoli cell in phenotype, we detected a number of markers for primary human Sertoli cells using RT-PCR, Western blots and immunocytochemistry. RT-PCR revealed that the transcripts of *AR, BMP4, BMP6, GATA4, GDNF, SCF, SOX9* and *WT1* were expressed in the immortalized human cells (Figure 2A), which was comparable to the expression level of these genes in primary human Sertoli cells (Figure 2B). In contrast, the mRNA of *3β-HSD*, *SMA* and *VASA*, markers for Leydig cells, myoid cells and germ cells, respectively, was undetectable in these cells (Figure 2A). High level of *hTERT* was observed in the immortalized human cells (Figure 2A). Western blots showed that the proteins of SCF, GDNF, BMP4, WT1 and SOX9 were expressed in human hS1 cells (Figure 2C), whereas 3β-HSD, SMA and VASA were undetected in this cell line (Figure 2C). Notably, the levels of FSHR and AR were much higher in hS1 cells compared to primary human Sertoli cells (Figure 2C).

**Figure 3:**
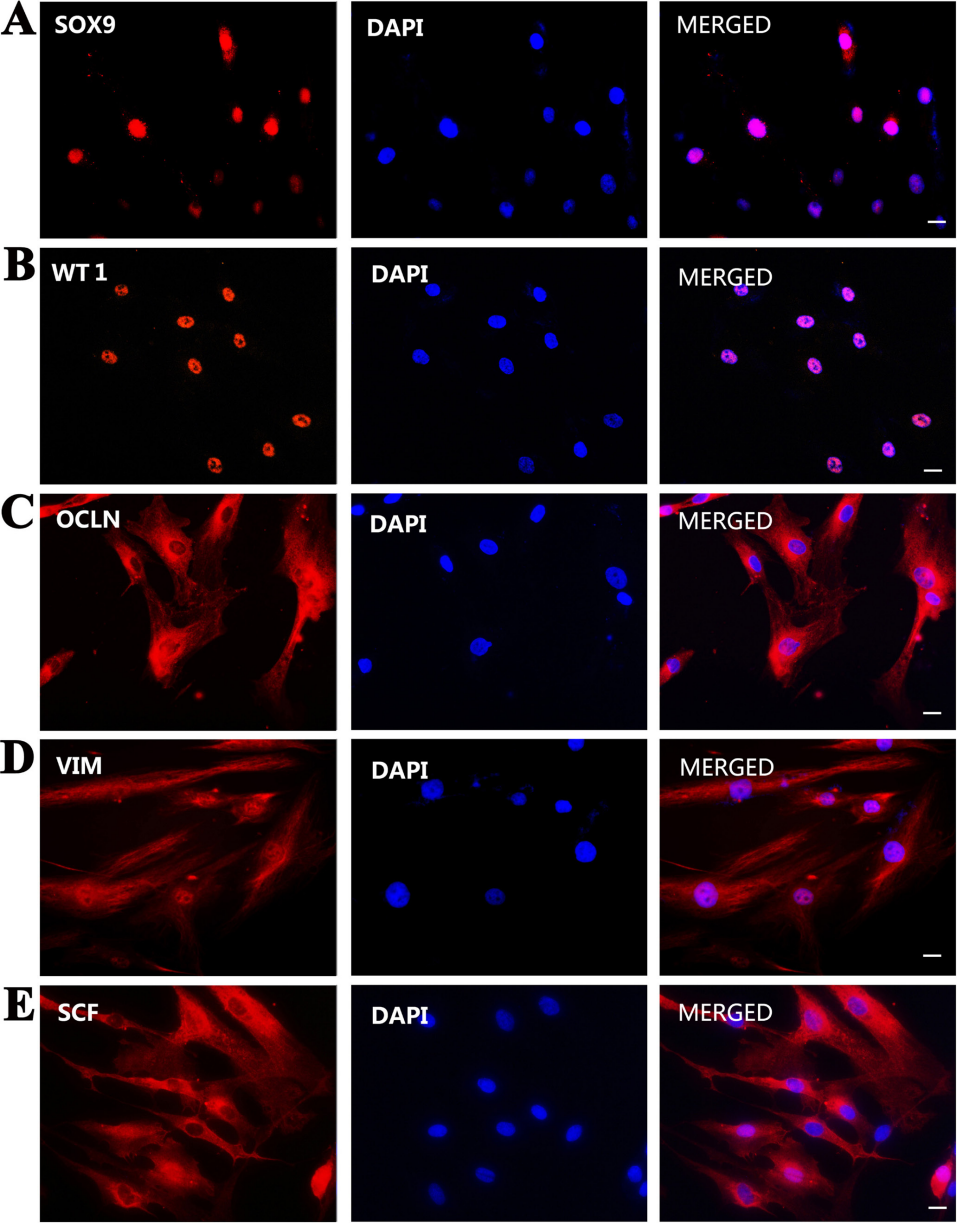
Phenotypic characteristics of the immortalized human Sertoli cells (**A–E**) Immunocytochemistry demonstrated the expression of SOX9 (A), WT1 (B), OCLN (C), VIM (D), and SCF (E) in the immortalized human Sertoli cells. Scale bars in A–E = 10 μm.

**Figure 4:**
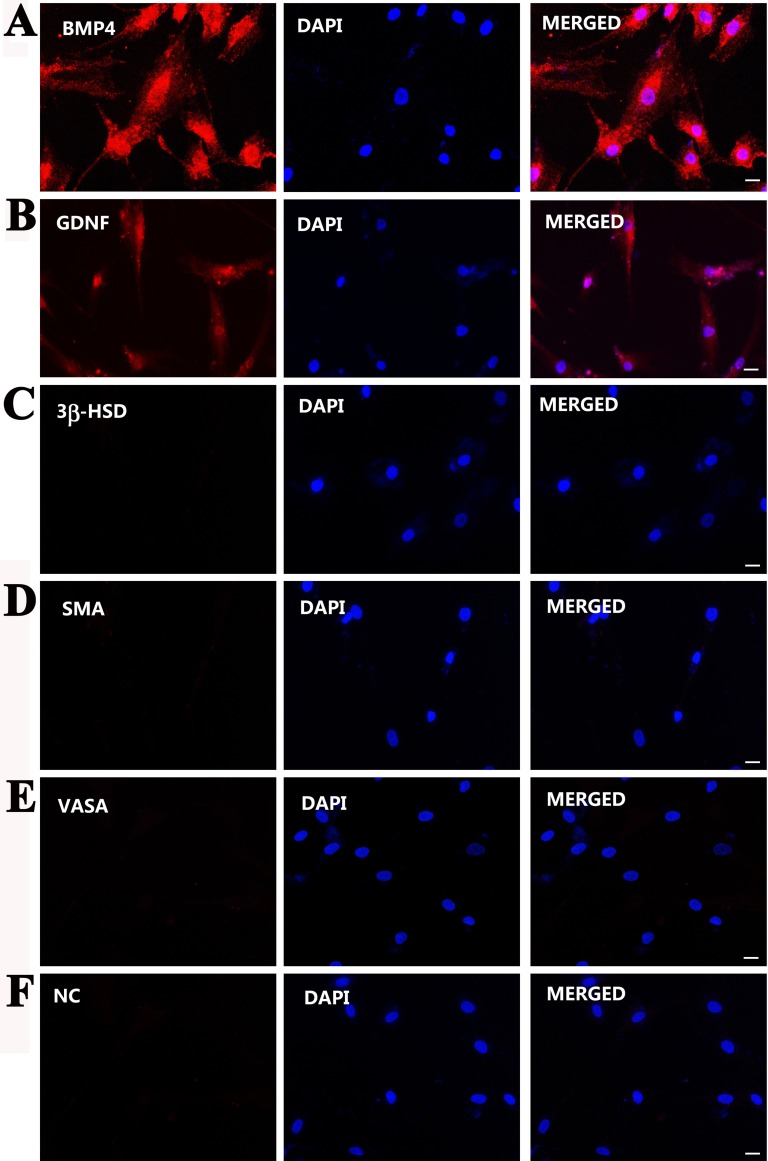
Phenotypic characterization of the immortalized human Sertoli cells (**A–F**) Immunocytochemistry displayed the expression of BMP4 (A), GDNF (B), 3β-HSD (C), SMA (D), and VASA (E) in the immortalized human Sertoli cells. Replacement of primary antibodies with isotype IgGs (F) served as negative controls (NC). Scale bars in A–F = 10 μm.

Furthermore, immunocytochemistry displayed that the immortalized cells were positive for SOX9 (Figure 3A), WT1 (Figure 3B), OCLN (Figure 3C), VIM (Figure 3D), SCF (Figure 3E), BMP4 (Figure 4A), GDNF (Figure 4B), but not for 3β-HSD (Figure 4C), SMA (Figure 4D), and VASA (Figure 4E). Replacement of primary antibodies with isotype IgGs was used as a negative control, and no immunnostaining was seen in these cells (Figure 4F), thus verifying specific expression of these proteins mentioned above in the immortalized cells.

### Similar global gene expression profiles between human Sertoli cell line and primary human Sertoli cells

**Figure 5:**
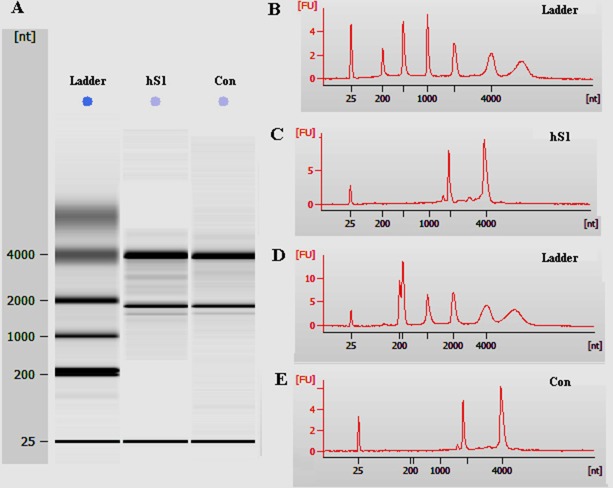
The quality assessment of total RNA of the immortalized human Sertoli cells and primary human Sertoli cells (**A**) Total RNA was quantified by the NanoDrop ND-2000 and the RNA integrity was assessed using Agilent Bioanalyzer 2100. (**B-E**) Electropherogram by Agilent bioanalyzer indicated the concentrations and nucleotides (nt) of RNA isolated from the immortalized human Sertoli cells (C) and primary human Sertoli cells (E). B and D were RNA ladders.

We next compared global gene expression profiling between the immortalized human cell line and primary human Sertoli cells using microarray analysis. Total RNA was quantified by the NanoDrop ND-2000 and RNA integrity was assessed using Agilent Bioanalyzer 2100. The total RNA from the immortalized human Sertoli cells and primary human Sertoli cells was of great quality, as evidenced by the RNA integrity number (RIN) values over 8.0 (Figure 5C, 5E, Table 1). In total, 24,118 genes were detected in both cell types by microarray analysis. There were 3,641 (15.0%) and 3,496 (14.5%) differentially expressed genes (up-regulated or down-regulated with 2.0 folds or more, respectively) between the immortalized human Sertoli cells and primary human Sertoli cells (Table 2). Thus, human Sertoli cell line had a 70.4% similarity of global gene profiles with primary human Sertoli cells. Microarray analysis showed that there was no significant change in the transcripts of *WT1*, *GATA4*, *GDNF*, *GATA1* and *SOX9* between the immortalized human Sertoli cells and primary human Sertoli cells (Table 3). The expression level of *BMP4*, *BMP6* and *FGF2* was higher in the immortalized human Sertoli cells than primary human Sertoli cells, while *LIF* mRNA was lower in the immortalized human Sertoli cells than primary Sertoli cells (Table 3).

**Table 1:** The quality of total RNA of the immortalized Sertoli cells (hS1) and primary human Sertoli cells (Control) by the NanoDrop ND-2000

Samples	Concentration	A260/280	A260/230	Volume(μl)	Quality	
	(μg/μl)				28S/18S	RIN
**Control**	0.0967	1.73	0.18	10	2	9
**hS1 cells**	0.1123	2.06	0.17	10	1.9	8.8

**Table 2:** The differently expressed gene profiles between the immortalized Sertoli cells and primary human Sertoli cells (Control)

Control	Human Sertoli cell line	Up-regulated	Down-regulated	Total genes	Similarity
Primary Sertoli cells	hS1 cells	3,641(15.0%)	3,496(14.5%)	24,118	70.4%

**Table 3:** The expression of representative genes between the immortalized Sertoli cells and primary human Sertoli cells (Control)

Gene Symbol	Primary Sertoli cell signal (normalized)	hS1 cell signal (normalized)	Log_2_ FC (hS1 vs. Control)
*GDNF*	2.7378905	4.001061	1.2631705
*BMP4*	7.070616	10.179245	3.108629
*BMP6*	2.913889	6.345799	3.43191
*LIF*	8.199608	9.237209	1.037601
*FGF2*	7.0719824	10.704937	3.6329546
*SOX9*	1.4255941	1.4340813	0.0084872
*WT1*	2.236927	2.0595062	–0.1774208
*GATA4*	10.017227	9.562365	–0.4548
*GATA1*	2.7784665	2.5375004	–0.2409661

**Figure 6:**
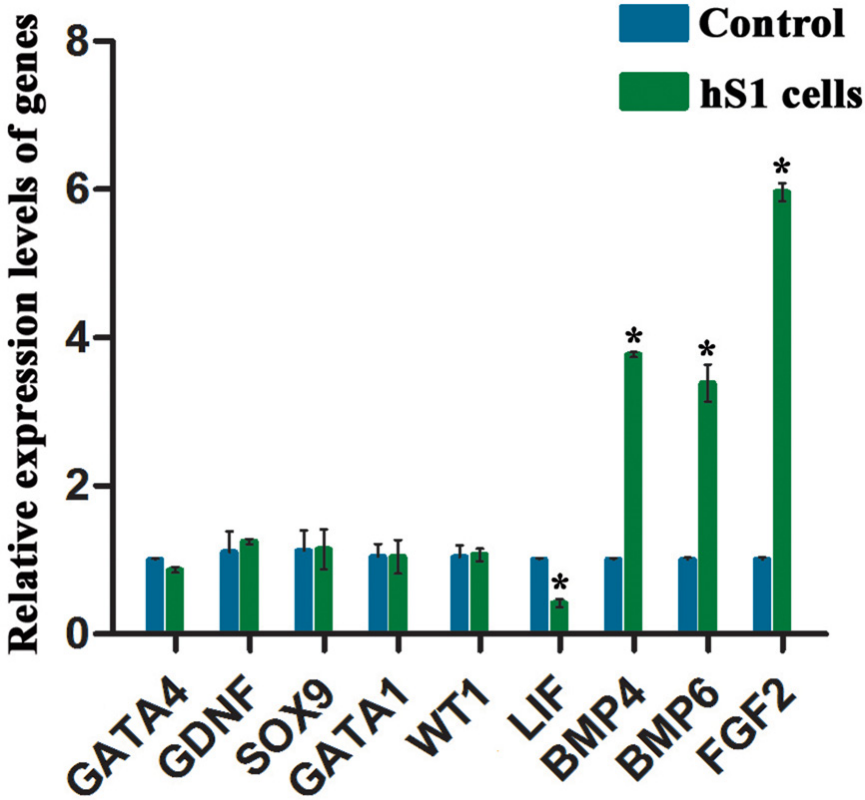
Expression levels of numerous genes between the immortalized human Sertoli cells and primary human Sertoli cells Real-time PCR demonstrated that the expression of *GATA4, GDNF, SOX9*, *GATA1, WT1, LIF, BMP4, BMP6*, and *FGF2* in the immortalized human Sertoli cells and primary human Sertoli cells. * indicated statistically significant differences (*p* < 0.05) between the immortalized human Sertoli cells and primary human Sertoli cells.

To confirm the results of microarray analysis, real-time PCR was performed to check the expression of numerous genes. We found that there was no significant difference in the expression of *GATA4*, *GDNF*, *SOX9*, *GATA1* and *WT1* between the immortalized human Sertoli cells and primary human Sertoli cells (Figure 6). The transcripts of *BMP4*, *BMP6* and *FGF2* were higher in the immortalized human Sertoli cells than primary human Sertoli cells, whereas *LIF* was expressed at a lower level in the immortalized human Sertoli cells compared to primary Sertoli cells (Figure 6). These results of real-time PCR were consistent with the data of our mRNA microarray.

### Proliferation potential of human Sertoli cell line

**Figure 7:**
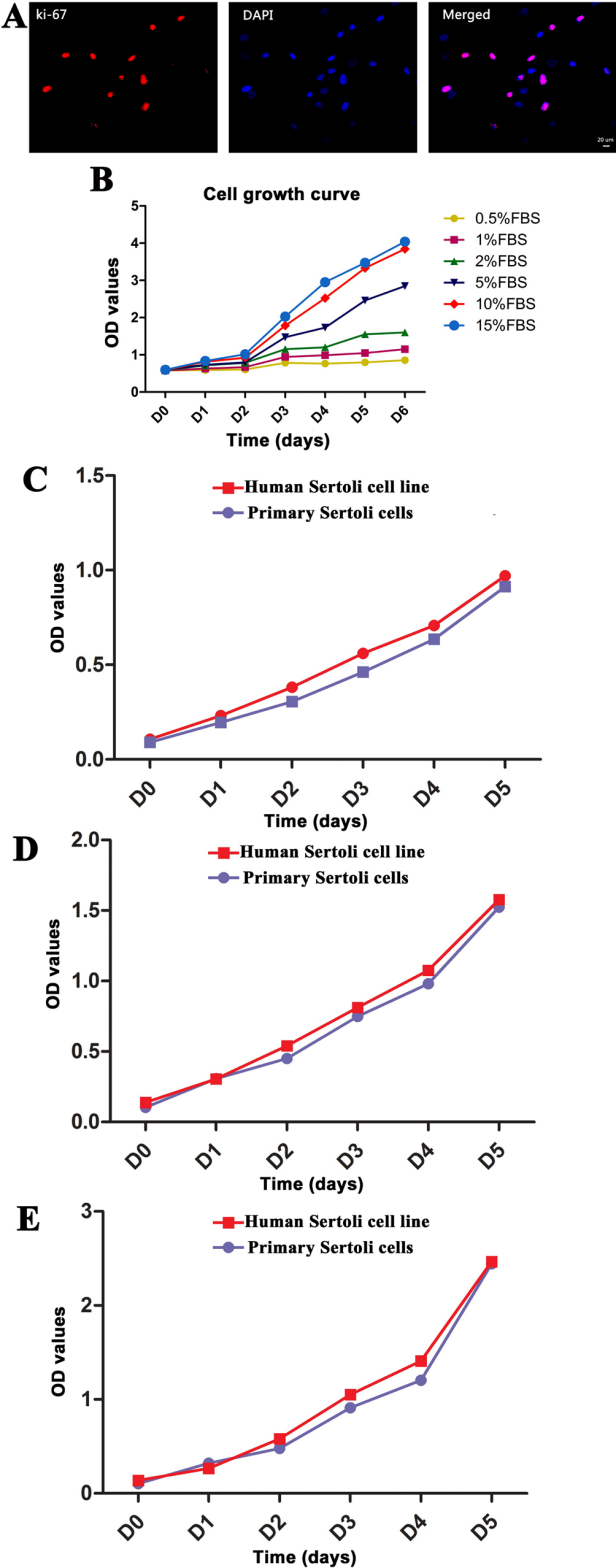
Proliferation capacity of the immortalized human Sertoli cells *in vitro* (**A**) Immunocytochemistry revealed the expression of Ki-67 in human Sertoli cell line. Scale bar in A = 20 μm. (**B**) CCK-8 assays were utilized to compare the effect of various concentrations of FBS ranging from 0.5% to 15% on the growth of human Sertoli cell line. (**C–E**) CCK-8 assays were employed to compare the proliferation of human Sertoli cell line and primary human Sertoli cells when they were cultured with 2% FBS (C), 5% FBS (D), and 10% FBS (E).

We utilized Western blots, immunocytochemistry, and CCK-8 assay to evaluate the proliferative potentials of human Sertoli cell line. Western blots showed similar expression level of PCNA protein between human Sertoli cell line and primary human Sertoli cells (Figure 2C). Immunocytochemistry revealed that more than 70% of human Sertoli cell line was positive for ki-67 (Figure 7A), suggesting this cell line had a high proliferation capacity.

We next checked the proliferative potentials of the immortalized human Sertoli cells under different concentrations of fetal bovine serum (FBS). CCK-8 assays showed that the proliferation of the immortalized human Sertoli cells was dependent on the concentrations of FBS. There was no obvious difference in the proliferation of the cell line when cultured in the medium supplemented with 0.5%–2% FBS, and 10% and 15% FBS had the optimal effect for promoting cell proliferation (Figure 7B). The doubling time of human Sertoli cell line was 1.3 days for culturing with 10% FBS and 1.03 days with 15% FBS (Figure 7B). Notably, human Sertoli cell line proliferate more rapidly than primary human Sertoli cells cultured with 2% FBS (Figure 7C), 5% FBS (Figure 7D), and 10% FBS (Figure 7E). Until now, the immortalized human Sertoli cells had been cultured for over 6 months in DMEM/F-12 with 10% FBS for more than 30 passages. During this long period of culture and different passages, this cell line didn't show morphological change or contact inhibition.

### Human Sertoli cell line assumed 90% of normal karyotype and excluded Y chromosome microdeletions

**Figure 8:**
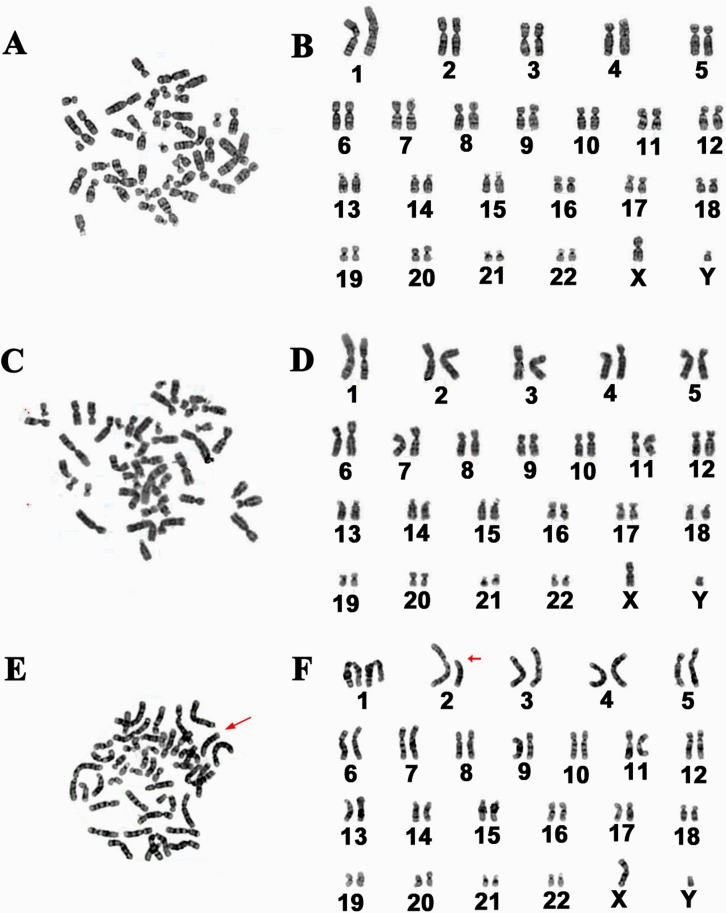
Karyotype analysis of the immortalized human Sertoli cells (**A–F**) Cytogenetic assay revealed normal karyotype (A–D) and abnormal karyotype (E–F) in the immortalized human Sertoli cells. The data were calculated from more than 100 cells of human Sertoli cell line.

Abnormal karyotype may occur in cell line due to transferring genes. We asked whether this alteration existed in human Sertoli cell line after overexpressing hTERT. Conventional cytogenetic analysis displayed that 90% of the immortalized human Sertoli cells had normal karyotype with 23 pairs of chromosomes (Figure 8A–Figure 8D), while about 10% of these cells showed abnormal karyotype with unbalanced translocation (Figure 8E–8F).

**Figure 9:**
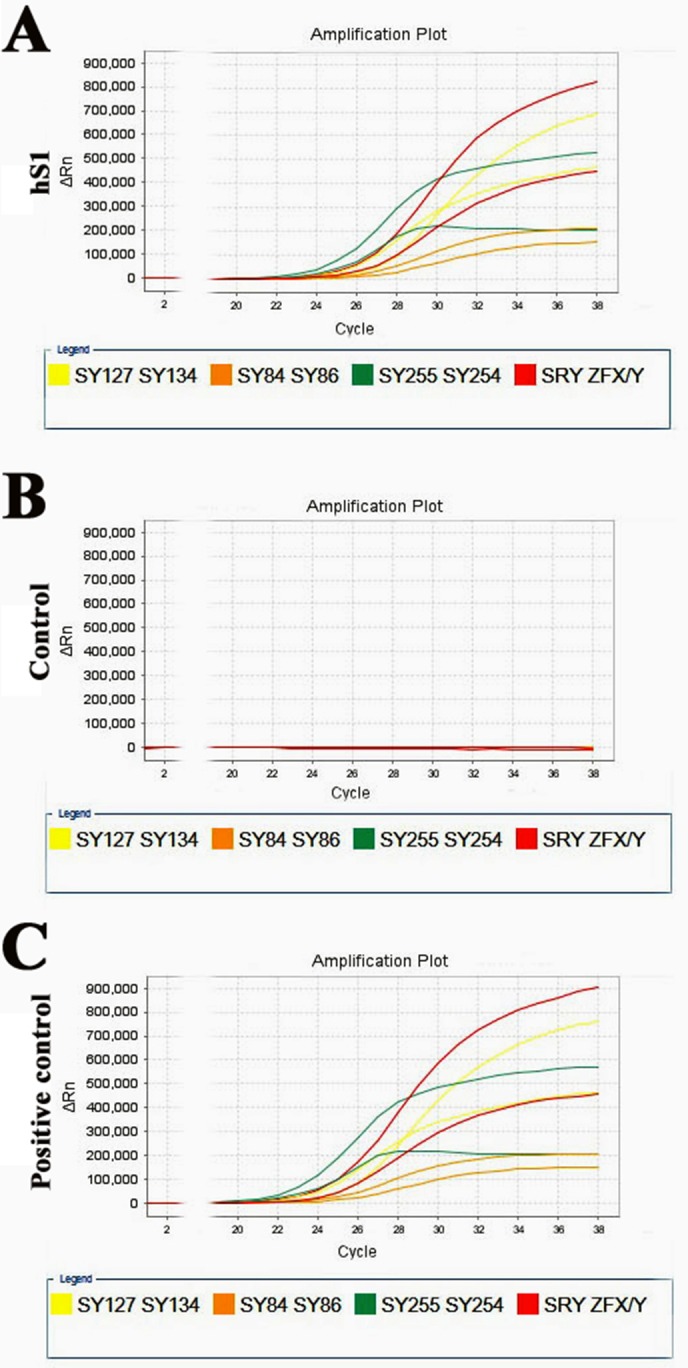
Y chromosome microdeletion analysis of the immortalized human Sertoli cells (**A–C**) Multiplex real-time PCR displayed the expression of all eight specific STS markers from AZFa, AZFb and AZFc regions, containing *ZFX*/*ZFY*, *SRY*, *sY254*, *sY127*, *sY86*, *sY134*, *sY84* and *sY255*, in the immortalized human Sertoli cells (A). DNA from normal human blood served as a positive control (C) and water substituted for DNA as a negative control (B).

Multiplex real-time PCR was used to assess whether there were Y microdeletions in the immortalized human Sertoli cells. As illustrated in Figure 9A, eight specific STS markers from AZFa, AZFb and AZFc regions, including *ZFX/ZFY*, *SRY*, *sY254*, *sY127*, *sY86*, *sY134*, *sY84* and *sY255*, were all detected in the immortalized human Sertoli cells, implicating that this cell line excludes Y chromosome microdeletions.

### Human Sertoli cell line didn't form tumors in xenografting mice

**Figure 10:**
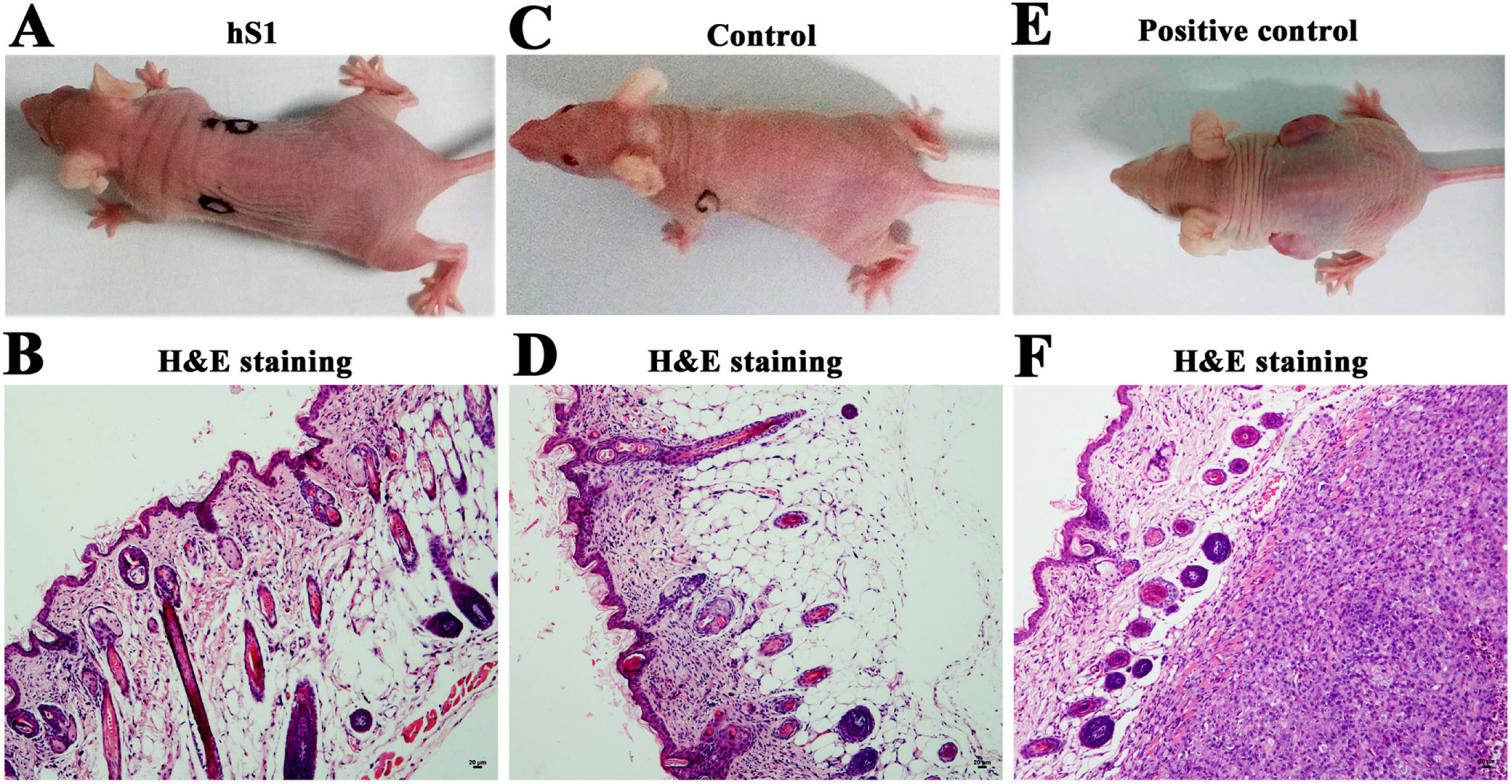
Tumorgenesis of the immortalized human Sertoli cells by nude mouse xenografting assays (**A-F**) Digital camera and H&E staining revealed no tumor formation in recipient mice with transplantation of the immortalized human Sertoli cells (**A, B**) or without cell transplantation (**C, D**). Tumor formation was observed in the recipient mice with transplantation of PC3 cells (E, F). Scale bars in B, D, F= 20 μm.

We further asked whether the immortalized human Sertoli cells had the ability to form tumors. Thirty nude mice were utilized for xenotransplantation assay *in vivo*. After 8 weeks of cell transplantation, no tumor formation was observed at each skin site of recipient mice (Figure 10A). H&E staining showed that there was regular and normal structure in the skin sites of transplantation (Figure 10B). In parallel, no tumor formation was seen in recipient mice without cell transplantation (Figure 10C–10D). In contrast, the tissue sections from skin of mice transplanted with PC3 cells displayed aberrant structure, and plenty of cancer cells occurred under the epidermis (Figure 10E–10F).

## DISCUSSION

Sertoli cells have great applications in basic research on uncovering mechanisms of human spermatogenesis and clinical usage. Although much progress has been made on genetic and epigenetic regulation of Sertoli cells in rodent spermatogenesis, very little is known about the roles and molecular mechanisms of human Sertoli cells in controlling the process of human spermatogenesis. This might be due to the facts that it is rather difficult to obtain human testis tissues and that primary human Sertoli cells have a limited proliferation capacity *in vitro*. Therefore, it is essential to establish a human Sertoli cell line to obtain sufficient cells for their usage from the bench to the bed side. There were several Sertoli cell lines that have been established in rodents [13, 16, 17]. Nevertheless, no human Sertoli cell line has yet been available. Here we have for the first time established a stable human Sertoli cell line by overexpressing hTERT. Immortalization of cells using TERT has certain advantages over the methods using the SV40 large T antigen or other approaches: i) TERT can immortalize cells without transformation or tumor formation [18]; and ii) cells immortalized by TERT not only proliferate but also differentiate or undergo maturation, whereas immortalized cells using SV40 large T antigen might lead to only proliferation without differentiation or maturation. We demonstrated that hTERT was stably expressed at both transcriptional and translational levels in human Sertoli cell line.

Significantly, human Sertoli cell line, namely hS1 cells, assumed the characteristics of primary human Sertoli cells. Firstly, this cell line had morphological features similar to primary human Sertoli cells when they were freshly separated by FACS and in culture. Secondly, human Sertoli cell line shared phenotypic characteristics with primary human Sertoli cells, since a number of markers for Sertoli cells, including GDNF, FGF2, BMP4, SCF, SOX9, GATA4, WT1, BMP6, and VIM, but not 3β-HSD, SMA and VASA (markers for Leydig cells, peritubular myoid cell and germ cells, respectively), were expressed in these cells. As a key component of the niche, Sertoli cells facilitate spermatogenesis by secreting a variety of growth factors and cytokines, e.g., GDNF, FGF2, BMP4, and SCF. All of these growth factors were detected in the human Sertoli cell line. It has been reported that GDNF and FGF2, produced by Sertoli cells, can promote the proliferation of spermatogonial stem cells from both humans and rodents [19–23]. GDNF, a member of transforming growth factor (TGF-β) super-family, is indispensable for the self-renewal of spermatogonial stem cells both *in vitro* and *in vivo* [22, 24]. BMP4, another member of the TGF-β superfamily, is secreted by Sertoli cells, and it stimulates the differentiation of spermatogonial stem cells by means of inducing KIT expression in the undifferentiated spermatogonia [25]. We have recently demonstrated that BMP4 regulates the proliferation of human Sertoli cells via Smad1/5 and ID2/3 pathway through an autocrine manner [26]. SCF, also produced by Sertoli cells, stimulates the proliferation and differentiation of type A spermatogonia through SCF/KIT pathway [14, 27]. SOX9 (sex-determining region Y box9 gene), GATA4 [28, 29] and WT1 [30] are essential for testis development. SOX9 can promote the maturity of Sertoli cells and the maintenance of spermatogenesis [31–33]. It has been reported that abortion of *WT1* leads to gonadal agenesis owing to the failure of genital ridge development [34]. Further studies unveil that Sertoli cell could be reprogramed into Leydig cell by deleting WT1. On the contrary, Leydig cell could express Sertoli cell markers by means of overexpressing WT1 [35]. Similar to *WT1*, deletion of *Gata4* in Sertoli cells results in complete loss of germ cells [36]. In addition, the connection between Sertoli cells and germ cells depend on VIM to a large extent, and exposure to dibutyl phthalate could result in aberrant vimentin cytoskeleton and abnormal spermatogenesis [37, 38]. Notably, the expression of GDNF, SCF, BMP4, SOX9, WT1 and VIM proteins in human Sertoli cell line was comparable to primary human Sertoli cells, suggesting that human Sertoli cell line might have similar function with primary human Sertoli cells in regards to the regulation of human male germ cells.

AR and FSHR, mature markers for adult Sertoli cells, are the two vital receptors expressed exclusively in Sertoli cells within seminiferous tubules, by which follicular stimulating hormone (FSH) and testosterone control the spermatogenesis [13, 39, 40]. AR-knockout mice are infertile and display abdominally located testes and developmental arrest of spermatocytes, round and elongated spermatids [6, 41, 42], implicating that AR-deficient Sertoli cells block spermatogenesis by prohibiting Sertoli cell maturation. Significantly, we found that the levels of FSHR and AR were much higher in human Sertoli cell line than primary human Sertoli cells, reflecting that human Sertoli cell line has some advantages over primary human Sertoli cells due to its more maturation for the usage in basic studies and clinical applications. In addition, the transcripts of *BMP4*, *BMP6*, and *FGF2* were higher in human Sertoli cell line than primary human Sertoli cells due to the overexpression of hTERT, which could be helpful for regulation of human spermatogenesis.

Human primary Sertoli cells are rare owing to the limited availability of human testis and the phenotype changes of Sertoli cells occur in culture with passages. It is worth noting that our human Sertoli cell line immortalized by hTERT has been cultivated *in vitro* for over 30 passages with no morphological or phenotypic changes. Furthermore, this human Sertoli cell line has great safety. It has been reported that immortalized cells exhibit ether structural or numerical chromosomal malformation by transducing SV40 or papillomaviruses E6 and E7 [43, 44]. We found that 90% of this human Sertoli cell line had normal karyotype. Additionally, neither Y chromosome microdeletions nor tumor formation was seen in human Sertoli cell line, implying vital applications of these cells in translational medicine.

## MATERIALS AND METHODS

### Acquisition of testicular biopsies from OA patients with normal spermatogenesis

Testicular biopsies were obtained from OA patients who underwent micro-dissection and testicular sperm extraction at Ren Ji Hospital, affiliated to Shanghai Jiao Tong University School of Medicine. Spermatogenesis was normal in all OA patients as evaluated by histology analysis of testis sections using hematoxylin and eosin (H&E) staining. The azoospermia of these patients were caused by inflammation or vasoligation rather than congenital absence of the vas deferens or other diseases. All experiments were conducted in accordance with relevant guidelines and regulations of the Institutional Ethical Review Committee of Ren Ji Hospital (license number of ethics statement: 2012–01), affiliated to Shanghai Jiao Tong University School of Medicine, and the written informed consents for testicular biopsies for research only were obtained by the patients.

### Isolation of human Sertoli cells from OA patients

Testicular tissues from OA patients were washed three times aseptically in Dulbecco modified Eagle medium Nutrient Mixture F-12 (DMEM/F-12) (Gibco) containing antibiotics with penicillin and streptomycin (Gibco). Seminiferous tubules were separated from testis biopsies by the first enzymatic digestion containing 2 mg/ml collagenase IV (Gibco) and 1 μg/ml DNase I (Roche) in 34°C water bath for 15 min. Sertoli cells and human male germ cells were obtained from seminiferous tubules using a second enzymatic digestion comprising 4 mg/ml collagenase IV, 2.5 mg/ml hyaluronidase (Sigma), 2 mg/ml trypsin (Sigma) and μg/ml DNase I and followed by differential plating pursuant to the protocol as previously described [45]. Cell suspension was seeded into polystyrene-treated dishes (Corning) in DMEM/F-12 supplemented with 10% FBS (Gibco) and incubated at 34°C in 5% CO_2_ for 1 day. In this case, suspending male germ cells and other types of cells were removed, and the adherent cells were Sertoli cells.

### Immortalization of human Sertoli cells

The expression vector, namely Lv-EF1A-hTERT-IRES-EGFP (Figure 1B), was purchased from Sidansai Biotechnology CO. LTD (Shanghai, China). Lenti-virus was packed by co-transfecting three plasmids, including VSVG, Δ8.9 and plasmid carrying gene *hTERT*, into 293T cells with lipofectamine^®^ 3000 (Invitrogen) according to the manufacturer's instruction. The titer was about 10^6^ TU/ml by infecting 293T cells with the packed lenti-virus. Human Sertoli cells were seeded in 6-well plates at 60% confluence, and the medium was removed and replenished with fresh DMEM/F-12 supplemented with 10% FBS containing 100 μl lenti-virus and 8 μg/ml polybrene (Sigma), and the cells were incubated at 37°C in 5% CO_2_ overnight. Twenty-four hours later, medium was changed with fresh DMEM/F-12 and 10% FBS, and the expression of EGFP was detected under a fluorescence microscope (Nikon) at 48 hours after infection. The immortalized human Sertoli cells, namely the EGFP-positive cells, were sorted by fluorescence-activated cell sorting (FACS) and cultured with DMEM/F-12 supplemented with 10% FBS in 5% CO_2_.

### RNA extraction, reverse transcription-polymerase chain reaction (RT-PCR) and real-time PCR

Total RNA was extracted from the immortalized human Sertoli cells at passage 2 (P2) using Trizol (Invitrogen) followed by the treatment of DNase I to remove potential contamination of genomic DNA. The cDNAs were synthesized using the RevertAid First Strand cDNA Synthesis Kit (Thermo Scientific) pursuant to the manufacturer's instruction. The primers of the chosen genes, including *GATA4* (GATA binding protein 4), *WT1*, *SOX9* (Sex Determining Region Y-Box9), *GDNF*, *BMP4*, SCF, *FSHR* (follicle-stimulating hormone receptor), *AR* (androgen receptor), 3β-HSD (hydroxy-delta-5-steroid dehydrogenase, 3 beta), *SMA* (alpha-smooth muscle actin), *VASA* (DEAD-box helicase 4, also called DDX4) and *GAPDH* (glyceraldehyde-3-phosphate dehydrogenase), were designed and listed in Table 4. The PCR reactions started at 94°C for 5 min and were performed as follows: denaturation at 95°C for 30 sec, annealing at 55–60°C for 30 sec as listed in Table 4, and elongation at 72°C for 30 sec, for 35cycles. After amplification, the samples were incubated for another 5 min at 72°C. PCR products were separated by electrophoresis in 1.5% agarose gels and visualized with ethidium bromide. Images were recorded and band intensities were analyzed using chemiluminescence (ChemiDoc^TM^ XRS^+^, Bio-Rad). Total RNA without RT (RT-) but with PCR using *GAPDH* primers served as negative controls.

**Table 4:** Primer sequences of genes used for RT-PCR

Genes		Primer sequences	Products size (bp)	Tm (°C)
*GATA4*	Forward	GCCTCCTCTGCCTGGTAAT	120	60
	Reverse	CAGTCCCATCAGCGTGTAAA		
*WT1*	Forward	TGACTCTCCACTCCTCCTCAC	115	60
	Reverse	ACCAACTCTTCCAGGCACAC		
*SOX9*	Forward	AGGTGCTCAAAGGCTACGACTG	322	60
	Reverse	TGCCCGTTCTTCACCGACT		
*GDNF*	Forward	GAAGTTATGGGATGTCGTG	419	58
	Reverse	TCAGTTCCTCCTTGGTTTC		
*SCF*	Forward	GTCATTGTTGGATAAGCGAGAT	457	60
	Reverse	ATGGCTGCCCAGTGTAGG		
*BMP4*	Forward	TTTGTTCAAGATTGGCTGTC	324	60
	Reverse	AGATCCCGCATGTAGTCC		
*AR*	Forward	CCTTCACCAATGTCAACTCC	198	60
	Reverse	CCACTGGAATAATGCTGAAGAG		
*BMP6*	Forward	AGCAATCTGTGGGTTGTGACT	228	60
	Reverse	GGTAGAGCGATTACGACTCTGTT		
*3β-HSD*	Forward	GCCGATTCCTTTCTGCTAGTAT	378	58
	Reverse	TGACTATGTGGCGGTTGAAG		
*SMA*	Forward	GATCTGGCACCACTCTTTCTAC	479	58
	Reverse	CAGGCAACTCGTAACTCTTCTC		
*VASA*	Forward	GCAGAAGGAGGAGAAAGTAGTG	289	60
	Reverse	CTCGTCCTGCAAGTATGATAGG		
*hTERT*	Forward	GGTGAACTTCCCTGTAGAAGAC	374	60
	Reverse	GGTTCTTCCAAACTTGCTGATG		
*GAPDH*	Forward	AATCCCATCACCATCTTCC	382	58
	Reverse	CATCACGCCACAGTTTCC		

Real-time PCR were performed using Power SYBR^®^ Green PCR Master Mix (Applied Biosystems) and a StepOnePlus Real-Time PCR System (Applied Biosystems) according to the manufacture's instruction. To quantify the PCR products, the 2^-ΔΔCt^ method was used in terms of the protocol as described previously [46]. The threshold of cycle values was normalized against threshold values of human housekeeping gene *GAPDH*. The primer pairs of chosen genes were listed in Table 5.

**Table 5:** The primer sequences of genes used for real-time PCR

Genes		Primer sequences	Tm (°C)
*BMP6*	Forward	AGCAATCTGTGGGTTGTGACT	60
	Reverse	GGTAGAGCGATTACGACTCTGTT	
*BMP4*	Forward	TAGCAAGAGTGCCGTCATTCC	60
	Reverse	GCGCTCAGGATACTCAAGACC	
*SOX9*	Forward	AGCGAACGCACATCAAGAC	60
	Reverse	CTGTAGGCGATCTGTTGGGG	
*GATA1*	Forward	TTGTCAGTAAACGGGCAGGTA	60
	Reverse	CTTGCGGTTTCGAGTCTGAAT	
*FGF2*	Forward	AGTGTGTGCTAACCGTTACCT	60
	Reverse	ACTGCCCAGTTCGTTTCAGTG	
*GATA4*	Forward	GCCTCCTCTGCCTGGTAAT	60
	Reverse	CAGTCCCATCAGCGTGTAAA	
*WT1*	Forward	TGACTCTCCACTCCTCCTCAC	60
	Reverse	ACCAACTCTTCCAGGCACAC	
*LIF*	Forward	CCAACGTGACGGACTTCCC	60
	Reverse	TACACGACTATGCGGTACAGC	
*GDNF*	Forward	GGCAGTGCTTCCTAGAAGAGA	60
	Reverse	AAGACACAACCCCGGTTTTTG	
*GAPDH*	Forward	CAGGAGGCATTGCTGATGAT	60
	Reverse	GAAGGCTGGGGCTCATTT	

### Immunocytochemistry of the immortalized human Sertoli cells

For immunocytochemical staining, the immortalized human Sertoli cells were fixed with 4% paraformaldehyde (PFA) for 15 min, washed three times with phosphate-buffered saline (PBS) for 5 min each and permeabilized in 0.5% triton-X 100 (Sigma) for 20 min. After washing with PBS, the cells were blocked in 3% serum or BSA for 60 min and followed by incubation with primary antibodies, including anti-SOX9 (Millipore), anti-WT1 (Santa Cruz), anti-OCLN (Abcam), anti-VIM (vimentin, Santa Cruz), anti-SCF (Sigma), anti-BMP4 (Abcam), anti-GDNF (Abcam), anti-3β-HSD (Santa Cruz), anti-VASA(Santa Cruz), and anti-Ki-67 (BD Biosciences), overnight at 4°C. Replacement of primary antibodies with isotype IgGs (Santa Cruz) served as negative controls. After washing three times in PBS for 10 min each, the cells were incubated with the secondary antibody (Sigma) conjugated with rhodamine at a 1:200 dilution for 1 hour at room temperature. DAPI (4, 6-diamidino-2-phenylindole, Sigma) was used to counterstain the nuclei, and images were captured with a fluorescence microscope (Nikon).

### Western blots

The immortalized human Sertoli cells and human primary Sertoli were lysed with RIPA buffer (Santa Cruz) for 30 min on ice. Cell lysates were cleared by centrifugation at 12,000 g for 10 min at 4°C, and the concentrations of total proteins were measured by BCA kit (Dingguo Company, China). Thirty micrograms of cell lysates from each sample were used for SDS-PAGE, and Western blots were performed according to the protocol as described previously [24]. The chosen antibody included anti-hTERT (Santa Cruz), anti-FSHR (Abcam), anti-AR (Santa Cruz), anti-GDNF, anti-SCF, anti-BMP4, anti-WT1, anti-SOX9, anti-PCNA (proliferating cell nuclear antigen, Santa Cruz), anti-3β-HSD, anti-VASA, anti-SMA, and anti-ACTB (Proteintech^TM)^. Replacement of primary antibodies with PBS served as negative controls (NC). After extensive washes in TBST, the images were detected by chemiluminescence (ChemiDoc^TM^ XRS^+^, Bio-Rad).

### Microarray analysis

Total RNA was extracted from immortalized human Sertoli cells at passage 15 (P15) and primary Sertoli cells using Trizol reagent (Invitrogen). Total RNA was quantified by the NanoDrop ND-2000 (Thermo Scientific) and the RNA integrity was assessed using Agilent Bioanalyzer 2100 (Agilent Technologies). MRNA microarrays were applied on Agient Human microarray 8*60 array (Ouyi, Shanghai, China), and the results were confirmed by real-time PCR. Briefly, total RNA were transcribed to double strand cDNA, synthesized into cRNA and labeled with Cyanine-3-CTP. The labeled cRNAs were hybridized onto the microarrays. After extensive washes, the arrays were scanned by the Agilent Scanner G2505C (Agilent Technologies). Feature Extraction software (version10.7.1.1, Agilent Technologies) was used to analyze array images to get raw data. Genespring software (version13.1, Agilent Technologies) was employed to perform the basic analysis with the raw data. The significantly differentially expressed genes were selected according to the criteria: *P value* < 0.05, Fold change ≥ 2.

### CCK-8 assays

Human immortalized Sertoli cells at passage 6 (P6) and primary human Sertoli cells were seeded in 96-well plates at a concentration of 2,000 or 1,000 cells per well in 100 μl DMEM/F-12 medium containing 0.5%, 1%, 2%, 5%, 10% and 15% of FBS respectively, and incubated at 37°C in 5% CO_2_ for 7 days. Cellular proliferation was measured every 24 hours according to the protocol of the Cell Counting Kit-8 (CCK-8) Kit (Dojin Laboratories).

### Karyotyping assays

Chromosomal karyotype analysis of the exponentially growing immortalized human Sertoli cells at passage 15 (P15) was conducted pursuant to the procedure described previously [12]. In brief, cells were inhibited with 5 μg/ml colchicine for 3 hours, followed by hypotonic treatment with 0.075 M KCl solution, fixed with 3:1 methanol-glacial acetic acid, and dropped onto chilled slides. Cells were stained with Giemsa and counted under a microscope. The recommendation of the International System for Human Cytogenetic Nomenclature was applied to analyze the karyotypes.

### Multiplex real-time PCR analysis

Multiplex real-time PCR (Tellgen Corporation, Shanghai) was utilized to assess whether Y microdeletions existed in the immortalized human Sertoli cells at passage 20 (P20) according to the manufacturer's instructions. Three different regions, AZFa, AZFb and AZFc, were analyzed with six specific sequence-tagged site (STS) markers, including *sY254*, *sY127*, *sY86*, *sY134*, *sY84* and *sY255*. Zinc finger protein, X-linked (ZFX)/zinc finger protein, Y-linked (ZFY), and sex determining region Y (SRY), were used as internal controls. DNA from normal human blood served as positive controls and water without DNA with all primers was used as negative controls.

### Tumor formation of the immortalized human Sertoli cells by xenotransplantation assays

Thirty male nude mice (nu/nu BALB-c) at 6-week-old were used for *in vivo* tumor-formation potential of the immortalized human Sertoli cells at passage 20 (P20). The mice were housed in constant laboratory conditions of a 12-hour light, 12-hour dark cycle and pathogen-free conditions and fed with water and food *ad libitum*. All mice were treated in accordance with the animal care and use guidelines of Ren Ji Hospital animal care and ethics review committee. For xenograft study, the mice were divided into three groups. In the first group, each nu/nu nude mouse was injected subcutaneously into the left and right posterior axillary fossa without cells in a total volume of 100 μl DMEM/F-12 and Matrigel (1:1). In the latter two groups, each nu/nu nude mouse was injected subcutaneously with 2 × 10^6^ immortalized human Sertoli cells or 2 × 10^6^ human prostate cancer cells PC3 suspended with 100 μl DMEM/F-12 and Matrigel (1:1). Two weeks later, the tissues from mouse transplanted sites were fixed in paraformaldehyde overnight, embedded in paraffin, and sectioned at 5 μm thickness. The sections were stained with H&E and observed under a microscope (Nikon).

### Statistical analysis

All data were presented as mean ± SEM from at least three independent experiments and analyzed by Student's *t-test* or one-way ANOVA with the appropriate post-hoc tests (Dunnet's test or Turkey's multiple comparison) using Prism (version 5, GraphPad Software), and *p* < 0.05 was considered statistically significant.

## SUMMARY

In conclusion, we have established the first human Sertoli cell line with the morphological and phenotypic attributes of primary human Sertoli cells. This human cell line can facilitate our understanding the biology of human Sertoli cells. Notably, this human Sertoli cell line has almost normal karyotype, excludes Y chromosome microdeletions and doesn't form tumor. Moreover, the unlimited proliferative capacity of this cell line ensures us to obtain adequate source of human Sertoli cells for uncovering their roles in regulating the complex human spermatogenesis and the treatments for male infertility as well as other human diseases.
